# Dataset in characterization of the polymer produced using different method of synthesis polychloromethylstyrene (PCMS) with clay and without clay

**DOI:** 10.1016/j.dib.2021.106738

**Published:** 2021-01-09

**Authors:** Nabilah Ismail, Nur Khairunnisa Nazri, Aidil Adhha Abdullah, Wan Mohd Norsani Wan Nik, Leonard James Wright

**Affiliations:** a Universiti Malaysia Terengganu, Terengganu, Malaysia; b University of Auckland, New Zealand

**Keywords:** Polychloromethylstyrene (PCMS), Clay Cloisite, Film, Spectra

## Abstract

Polychloropolymethylstyrene (PCMS) polymers were synthesized with clay Cloisite and without clay Cloisite and chloromethylstyrene (CMS) combine with styrene (1:1) v/v or known as copolymer and clay Cloisite by the polymerization process. The attenuated total reflection-Fourier transform infrared (ATR-FTIR) spectra of each polymer synthesized are reported. The spectra of IR shows the different value of the wavenumber and intensity for each set of different sample. The spectra can be as a reference for others to use in synthesizing this polymer and clay Cloisite for different type of application.

## Specifications Table

**Table:** 

Subject	Chemistry (General)
Specific subject area	Synthesis of the polymer
Type of data	Different methods of synthesis of polychloromethylstyrene (PCMS) and figure of FTIR Spectra
How data were acquired	Attenuated total reactance Fourier transform infrared (ATR FTIR) spectra was performed using Fourier Transform Infrared Spectrophotometer IR Tracer-100 Solution Shimadzu.
Data format	Raw Data after synthesized and after the formation of the film
Parameters for data collection	4-Vinylbenzylchloride were synthesized by specific procedure with different clays Cloisite, different ratio and type of form (flakes and film)
Description of data collection	The ATR-FTIR instrument was operated with KBR detector and KBR beam splitter were generated from 40 wavenumbers at the range of 4000 cm^−1^ to 400 cm^−1^ wavenumbers
Data source location	Universiti Malaysia Terengganu Terengganu Malaysia
Data Accessibility	With the article
Related research article	N. K. Nazri, N. Ismail, M. A. A. Abdullah, F. Hashim, L. J. Wright, Synthesis and characterization of catalytic polymer-clay film for treatment of 17 α-ethinylestradiol, Malaysian Journal of Analytical Sciences. *24*(3), 330–338.

## Value of the Data

• It is important to make superior/versatile polychloromethylstyrene (PCMS) polymer that has enhanced properties once cast as a film making the film more stable, more temperature resistance, and increasing the shelf life of the polymer.
• The researcher might use this data to form a polymer and clay film with a particular type of clay and its ratio.
• The researcher can use data to compare and design the experiment involving polymers such as CMS with clays.
• Data can be useful in producing different types of polymer derivatives or modifications using starting material CMS. This modified polymer can anchor or attract any desired compounds or target materials for further use.

## 1 Data Description

**Fig. 1:**
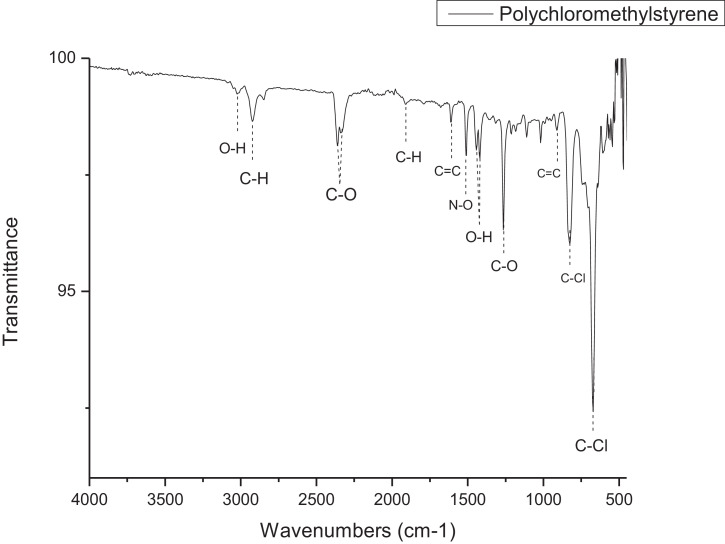
ATR-FTIR spectrum of polychloromethylstyrene (PCMS) flakes.

**Fig. 2:**
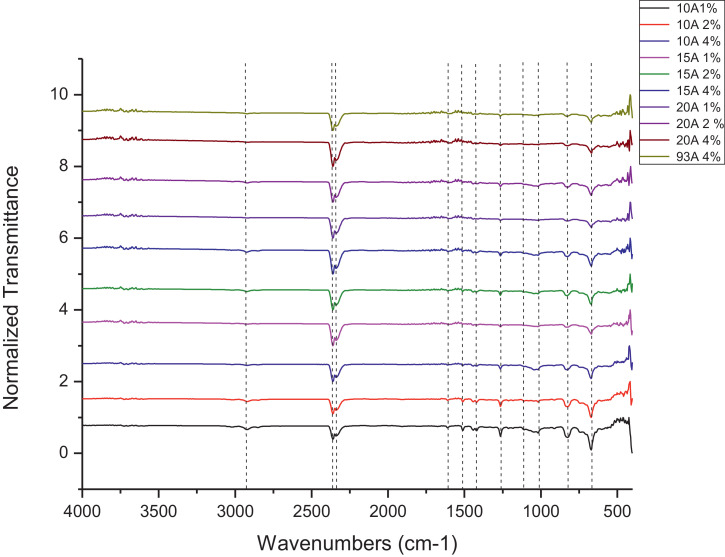
All synthesized PCMS with different types of clay and different percentage of clay.

**Fig. 3:**
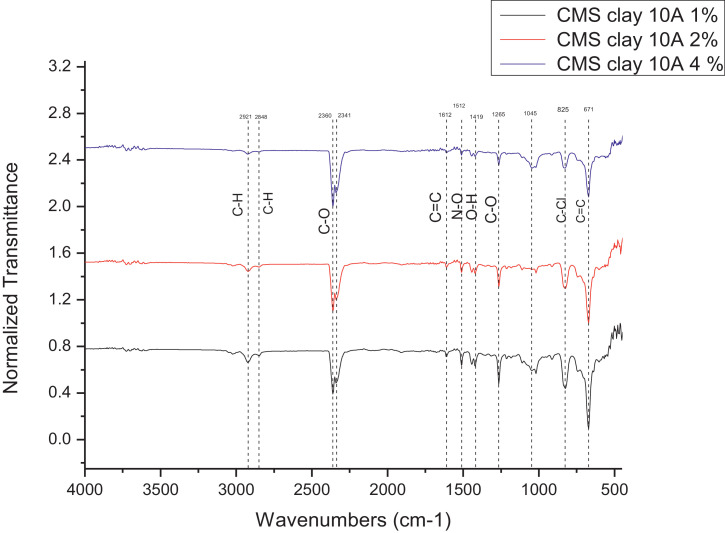
ATR-FTIR spectra of PCMS with clay Cloisite 10A.

**Fig. 4:**
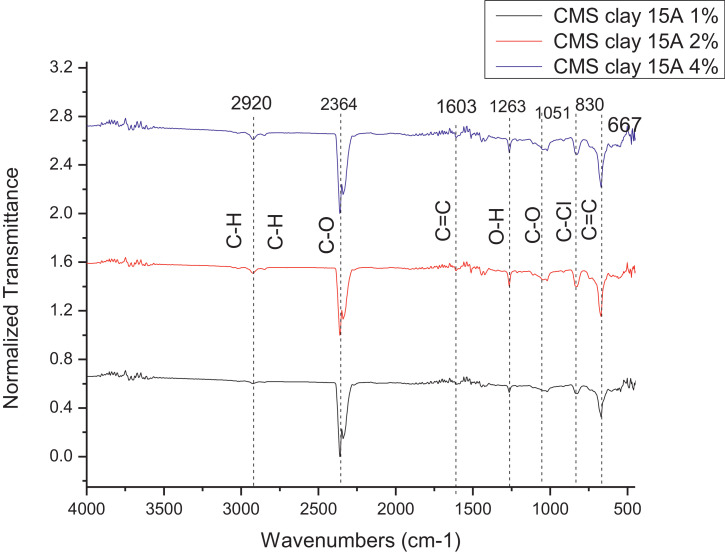
ATR-FTIR spectra of PCMS with clay Cloisite 15A.

**Fig. 5:**
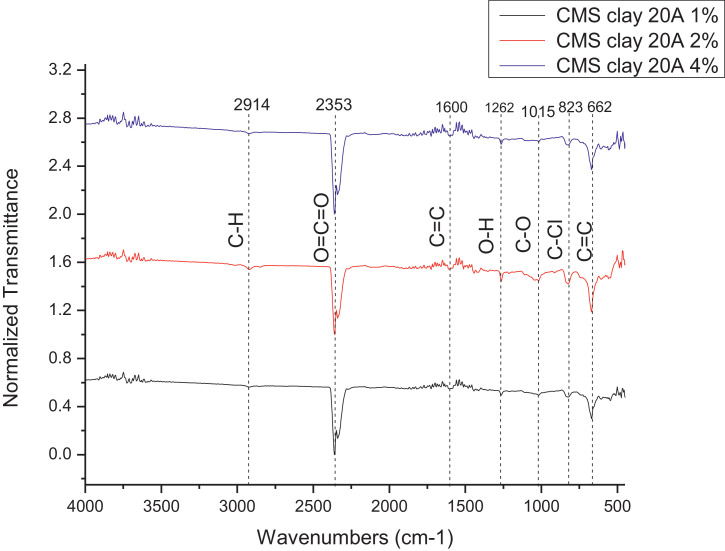
ATR-FTIR spectra of PCMS with clay Cloisite 20A.

**Fig. 6:**
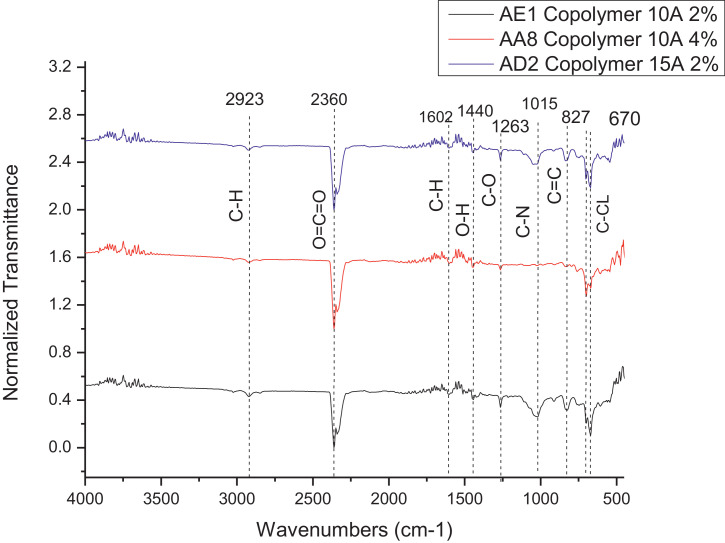
ATR-FTIR spectra of PCMS and styrene (copolymer) with clay Cloisite.

**Fig. 7:**
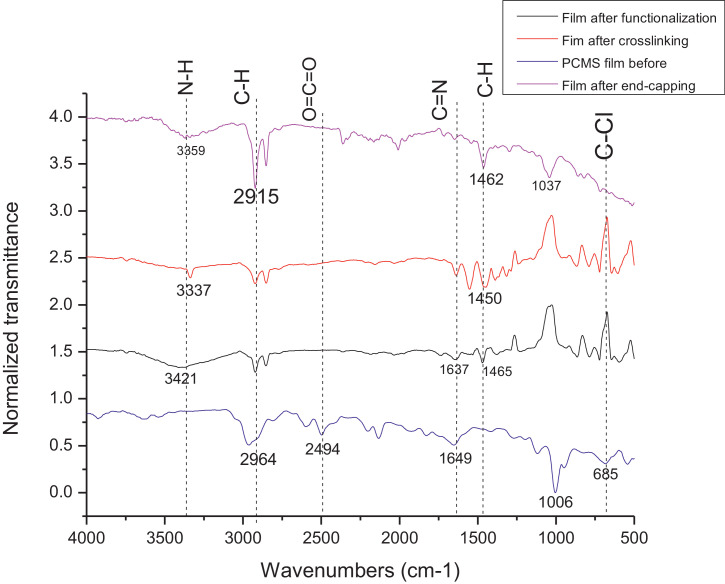
ATR-FTIR spectra of film PCMS and clay Cloisite 10A 4% before and after functionalized, crosslinking and end-capping process.

**Fig. 8:**
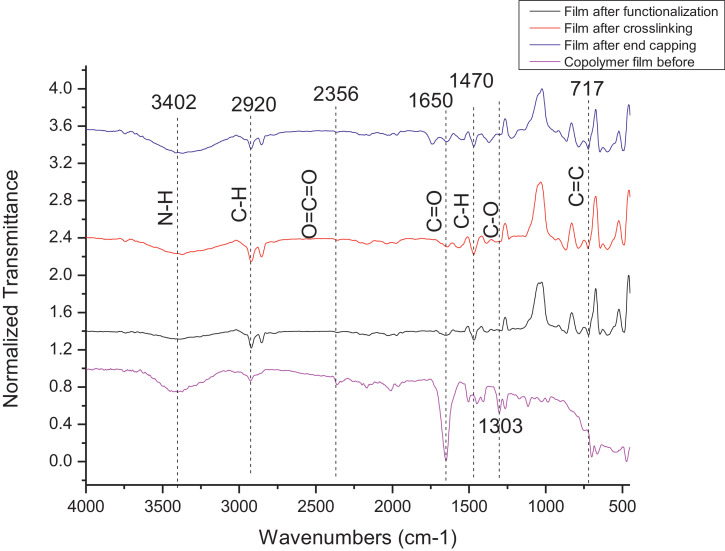
ATR-FTIR spectra of film PCMS with styrene (copolymer) and clay Cloisite 10A 4% before and after functionalized, crosslinking and end-capping process.

The dataset contains FTIR data of the polymer synthesized with a different combination of clay and its ratio. The monomer of chloromethylstyrene (CMS) was synthesized with and without combine with clay Cloisite. Clays and their modified forms have received significant interest for their use as adsorbents of metal ions from aqueous medium due to readily available and much cheaper than activated carbon, zeolites, and membranes [1]. The monomer of CMS was also synthesized with styrene with and without the presence of clay. Three different types of clay Cloisite were used which are 10A, 15A and 20A. Clay Cloisite is organically modified layered magnesium aluminum silicate platelets. 10A, 15A and 20A referred to the modifier (intercalatant) applied in the modification of montmorillonite (MMT). To observe which combination is better with the variation of the ratio of clay, these polymers were characterized using ATR-FTIR. Based on Table 1, the FTIR spectra of CMS without clay Cloisite and CMS with clay Cloisite 10A, 15A and 20A with different percentage of clay Cloisite used. This article provides experimental data of characterization of each of CMS synthesized. The different ratios used and different types of clay also used presence. Fig. 1 shows ATR-FTIR spectrum of polychloromethylstyrene (PCMS) flakes, which flakes do not contain any clay Cloisite and the raw data provided at supplementary data file FTIR_1. Fig. 2 shows the spectra combination of all synthesized PCMS with different types of clay and different percentage of clay and Fig. 3 shows ATR-FTIR spectra of PCMS with clay Cloisite 10A with the percentage of clay Cloisite 1%, 2% and 4% at supplementary data labeled FTIR_2. Besides, from the supplementary file FTIR_3, Fig. 4 shows ATR-FTIR spectra of PCMS with clay Cloisite 15A with different percentages of clay Cloisite and Fig. 5 ATR-FTIR shows the spectra of PCMS with clay Cloisite 20A 1%, 2% and 4% of clay Cloisite presence. Fig. 6 ATR-FTIR spectra of PCMS and styrene (copolymer) with clay Cloisite. Fig. 7 ATR-FTIR spectra of film PCMS and clay Cloisite 10A 4% before and after functionalized, crosslinking, and end-capping processes. Fig. 8 ATR-FTIR spectra of film PCMS with styrene (copolymer) and clay Cloisite 10A 4% before and after functionalized, crosslinking, and end-capping processes. All spectra show different intensities and different wavelengths. Supplementary data for Fig. 5, Fig. 6, Fig. 7 and Fig. 8 as attached at file FTIR_4, FTIR_5, FTIR 6 and FTIR_7 respectively.

**Table 1:** Type of samples characterized (homopolymerization).

Sample	Percentage of clay used (%)
CMS without clay Cloisite	–
CMS with clay Cloisite 10A	1
CMS with clay Cloisite 10A	2
CMS with clay Cloisite 10A	4
CMS with clay Cloisite 15A	1
CMS with clay Cloisite 15A	2
CMS with clay Cloisite 15A	4
CMS clay Cloisite 20A	1
CMS with clay Cloisite 20A	2
CMS with clay Cloisite 20A	4
CMS and styrene with clay Cloisite 10A	2
CMS and styrene with clay Cloisite 10A	4
CMS and styrene with clay Cloisite 15A	2

## 2 Experimental Design, Materials and Methods

### 2.1 Materials

CMS, styrene, clay Cloisite 10A, clay Cloisite 15A and clay Cloisite 20A, sodium hydroxide (NaOH), methyl ethyl ketone (MEK), methanol, dimethylformamide (DMF) and N-methyl pyrrolidone (NMP).

### 2.2 Methods

#### 2.2.1 Infrared spectroscopy

Fourier Transform Infrared Spectrophotometer IR Tracer-100 Solution Shimadzu was used to perform attenuated total reflectance Fourier transform infrared spectroscopy (ATR-FTIR). The number of scans is 40 scans which scan at 4000 to 400 cm^−1^ wavenumbers. The band positions were obtained using the IR Tracer Software.

#### 2.2.2 Data FTIR

*Synthesis of CMS without clay Cloisite*: Synthesis of polymer without solvent called (Homopolymerization)**.** 15 mL of CMS was mix with 0.5% NaOH (w/v) to remove inhibitor from the monomer and repeat three times. The monomer was washed with deionized water until the water becomes neutral after removed the inhibitor. To remove the excess water, potassium carbonate was added to the monomer. Heat the monomer at the temperature of 85 °C with a stirring speed of 150 rpm in nitrogen condition. The CMS was heated until completely viscous. The viscosity of the solution will change from runny-like water to honey-like viscosity. After viscous, MEK was dropped in the viscous solution and the mixture was dropped added slowly to the volume of 20 mL in the beaker that contained methanol to obtain the polymer flakes. The flakes were then filtered using a Buchner funnel and dried for 10 minutes in the oven [2]. ATR-FTIR; 3021, 2924, 2346, 1909, 1605, 1513, 1439,1418, 1264, 911, 825, 672 cm^−1^.

#### 2.2.3 Synthesis of polymer with clay Cloisite

The 15 mL CMS was removed the inhibitor first by adding 15 mL of 0.5% NaOH in the separating funnel and repeat for three times. Then, the deionized water was added in the separating funnel to wash the remaining NaOH. Sodium carbonate was added to remove excess water. An amount of clay Cloisite was added according to the percentage of clay needed into a flat bottom flask containing CMS. It was heated in an oil bath with a temperature under 85 °C with a stirring speed of 150 rpm in nitrogen condition. The CMS was heated until completely viscous. The viscosity of the solution will change from runny-like water to honey-like viscosity. After viscous, MEK was dropped in the viscous solution and the mixture was dropped added slowly to the volume of 20 mL in the beaker that contained methanol to obtain the polymer flakes. The flakes were then filtered using Buchner funnel and dried for 10 minutes in the oven.

**CMS with clay Cloisite 10A 1%**

The 1% (w/v) of the clay Cloisite 10A were synthesized as mentioned above. ATR-FTIR; 2921, 2848, 2360, 2341, 1612, 1512, 1419, 1265, 1045, 825, 671 cm^−1^.

**CMS with clay Cloisite 10A 2%**

The 2% (w/v) of the clay Cloisite 10A were synthesized as mentioned above. ATR-FTIR; 2921, 2848, 2360, 2341, 1612, 1512, 1419, 1265, 1045, 825, 671 cm^−1^.

**CMS with clay Cloisite 10A 4%**

The 4% (w/v) of the clay Cloisite 10A were synthesized as mentioned above. ATR-FTIR; 2921, 2848, 2360, 2341, 1612, 1512, 1419, 1265, 1045, 825, 671 cm^−1^.

**CMS with clay Cloisite 15A 1%**

The 1% (w/v) of the clay Cloisite 15A were synthesized as mentioned above. ATR-FTIR; 2920, 2364, 1603, 1263, 1051, 830, 667 cm^−1^.

**CMS with clay Cloisite 15A 2%**

The 1% (w/v) of the clay Cloisite 10A were synthesized as mentioned above. ATR-FTIR; 2920, 2364, 1603, 1263, 1051, 830, 667 cm^−1^.

**CMS with clay Cloisite 15A 4%**

The 4% (w/v) of the clay Cloisite 15A were synthesized as mentioned above. ATR-FTIR; 2920, 2364, 1603, 1263, 1051, 830, 667 cm^−1^.

**CMS with clay Cloisite 20A 1%**

The 1% (w/v) of the clay Cloisite 20A were synthesized as mentioned above. ATR-FTIR; 2914, 2353, 1600, 1015, 823, 662 cm^−1^.

**CMS with clay Cloisite 20A 2%**

The 1% (w/v) of the clay Cloisite 20A were synthesized as mentioned above. ATR-FTIR; 2914, 2353, 1600, 1015, 823, 662 cm^−1^.

**CMS with clay Cloisite 20A 4%**

The 1% (w/v) of the clay Cloisite 20A were synthesized as mentioned above. ATR-FTIR; 2914, 2353, 1600, 1015, 823, 662 cm^−1^.

#### 2.2.4 Synthesis of polymer and copolymer with clay Cloisite

15 mL of 0.5% NaOH was added to 15 mL CMS in separating funnel to remove inhibitor from CMS and 15 mL of 2.5% NaOH was added to 15 mL styrene and repeat three times to remove inhibitor. Then, the deionized water was added in the separating funnel to wash the remaining NaOH. Sodium carbonate was added to remove excess water. An amount of clay Cloisite was added according to the percentage of clay needed into flat bottom flask containing CMS. It was heated in an oil bath with a temperature of under 85 °C with a stirring speed of 150 rpm in nitrogen condition. The CMS was heated until completely viscous. The viscosity of the solution will change from runny-like water to honey-like viscosity. After viscous, MEK was dropped in the viscous solution and the mixture was dropped added slowly to the volume of 50 mL in the beaker that contained methanol to obtain the polymer flakes. The flakes were then filtered using Buchner funnel and dried for 10 minutes in the oven.

**Copolymer CMS and styrene with clay Cloisite 10A 2%**

The 2% (w/v) of the clay Cloisite 10A were synthesized with CMS and styrene as mentioned above. ATR-FTIR; 2923, 2360, 1602, 1440, 1263, 1015, 827, 670 cm^−1^.

**Copolymer CMS and styrene with clay Cloisite 10A 4%**

The 4% (w/v) of the clay Cloisite 10A were synthesized as mentioned above. ATR-FTIR; 2923, 2360, 1602, 1440, 1263, 1015, 827, 670 cm^−1^.

**Copolymer CMS and styrene with clay Cloisite 15A 2%**

The 2% (w/v) of the clay Cloisite 20A were synthesized as mentioned above. ATR-FTIR; 2923, 2360, 1602, 1440, 1263, 1015, 827, 670 cm^−1^.

**Formation of film CMS and clay Cloisite 10A 4%**

The 4% (w/v) of the clay Cloisite 10A and CMS were synthesized as mentioned above. After synthesized, the polymer flakes formed will be dissolved with DMF and were cast on the glass. After cast for 5 hours, the fil was cured in the oven for 5 hours. The functionalization was proceeded by heating the film in (N, N-dimethylhexadecylamine:1,4-dioxane) (6:4), crosslinked with (N, N-dimethylhexadecylamine: 1,6-diaminohexane) (6:4) and diethanolamine for 5 hours each. ATR-FTIR; 2964, 2494, 2128, 1649, 1006, 685. 3421, 2915, 1637, 1465. 3337, 2915, 1450.3359, 1915, 1462, 1037 cm^−1^.

**Formation of film CMS and styrene copolymer with clay Cloisite 10A 4%**

The 4% (w/v) of the clay Cloisite 10A and CMS were synthesized as mentioned above. After synthesized, the polymer flakes formed will be dissolved with NMP and were cast on the glass. After cast for 5 hours, the fil was cured in the oven for 5 hours. The functionalization was proceeded by heating the film in (N, N-dimethylhexadecylamine:1,4-dioxane) (6:4), crosslinked with (N, N-dimethylhexadecylamine: 1,6-diaminohexane) (6:4) and diethanolamine for 5 hours each. ATR-FTIR; 3402, 2920, 1650, 1470, 1303, 717. 3402, 2920, 1650, 1470, 717. 3402, 2920, 1650, 1470, 717. 3402, 2920, 1650, 1470, 717 cm^−1^ respectively.

## CRediT Author Statement

**Nur Khairunnisa**: Writing review and editing; **Nabilah Ismail**: Writing original draft; **Aidil Adhha Abdullah**: Validation; **Wan Mohd Norsani Wan Nik**: Supervision; **James Wright**: Conceptualization and Methodology.

## Declaration of Competing Interest

The authors declare that they have no known competing financial interests or personal relationships which have, or could be perceived to have, influenced the work reported in this article.

## Appendix Supplementary materials

Image, application 1

Image, application 2

Image, application 3

Image, application 4

Image, application 5

Image, application 6

Image, application 7

## Footnotes

Supplementary material associated with this article can be found in the online version at doi:10.1016/j.dib.2020.106631.
